# Machine learning for patient risk stratification for acute respiratory distress syndrome

**DOI:** 10.1371/journal.pone.0214465

**Published:** 2019-03-28

**Authors:** Daniel Zeiberg, Tejas Prahlad, Brahmajee K. Nallamothu, Theodore J. Iwashyna, Jenna Wiens, Michael W. Sjoding

**Affiliations:** 1 Computer Science and Engineering, University of Michigan, Ann Arbor, MI, United States of America; 2 Department of Internal Medicine, University of Michigan, Ann Arbor, MI, United States of America; 3 Institute for Healthcare Policy & Innovation, University of Michigan, Ann Arbor, MI, United States of America; 4 VA Center for Clinical Management Research, US Department of Veterans Affairs, Ann Arbor, MI, United States of America; 5 Michigan Integrated Center for Health Analytics and Medical Prediction, University of Michigan, Ann Arbor, MI, United States of America; 6 Institute for Social Research, University of Michigan, Ann Arbor, MI, United States of America; 7 Center for Computational Medicine and Bioinformatics, University of Michigan, Ann Arbor, MI, United States of America; Texas A&M University, UNITED STATES

## Abstract

**Background:**

Existing prediction models for acute respiratory distress syndrome (ARDS) require manual chart abstraction and have only fair performance–limiting their suitability for driving clinical interventions. We sought to develop a machine learning approach for the prediction of ARDS that (a) leverages electronic health record (EHR) data, (b) is fully automated, and (c) can be applied at clinically relevant time points throughout a patient’s stay.

**Methods and Findings:**

We trained a risk stratification model for ARDS using a cohort of 1,621 patients with moderate hypoxia from a single center in 2016, of which 51 patients developed ARDS. We tested the model in a temporally distinct cohort of 1,122 patients from 2017, of which 27 patients developed ARDS. Gold standard diagnosis of ARDS was made by intensive care trained physicians during retrospective chart review. We considered both linear and non-linear approaches to learning the model. The best model used L2-logistic regression with 984 features extracted from the EHR. For patients observed in the hospital at least six hours who then developed moderate hypoxia, the model achieved an area under the receiver operating characteristics curve (AUROC) of 0.81 (95% CI: 0.73–0.88). Selecting a threshold based on the 85^th^ percentile of risk, the model had a sensitivity of 56% (95% CI: 35%, 74%), specificity of 86% (95% CI: 85%, 87%) and positive predictive value of 9% (95% CI: 5%, 14%), identifying a population at four times higher risk for ARDS than other patients with moderate hypoxia and 17 times the risk of hospitalized adults.

**Conclusions:**

We developed an ARDS prediction model based on EHR data with good discriminative performance. Our results demonstrate the feasibility of a machine learning approach to risk stratifying patients for ARDS solely from data extracted automatically from the EHR.

## Introduction

Acute Respiratory Distress Syndrome (ARDS) is a common and devastating critical illness, developing in 23% of patients receiving invasive mechanical ventilation, and with a hospital mortality rate of 40% [1]. However, ARDS is frequently missed or diagnosed late by practicing clinicians. When patients with ARDS go unrecognized, they do not receive established ARDS treatments and suffer worse outcomes [1–4]. Thus, there is a critical need for risk stratification tools that can accurately identify high-risk patients early in their course of illness [5–7].

The most well-known ARDS risk stratification model is the Lung Injury Prediction Score (LIPS) [8]. The score utilizes a small set of clinical variables, including the presence of high-risk predisposing conditions (e.g., aspiration of gastric contents, sepsis, pneumonia), risk modifiers (e.g., previous alcohol abuse, recent chemotherapy, diabetes), and some specific vital sign abnormalities (e.g., respiratory rate > 30, oxygen saturation < 95%). Many of the clinical variables in LIPS require manual chart review by a physician or trained clinical researcher. As a result, the score cannot be easily automated and integrated into an electronic health record (EHR) system nor has it been widely implemented in clinical practice. In addition, though the model originally demonstrated good discriminative performance in its original multi-center validation study, with an AUROC = 0.80 [8], its performance dropped when applied to other populations [9, 10]. These performance limitations, in addition to the requirement of manual chart review, have limited the use of LIPS in both clinical research settings and clinical practice.

Accordingly, a prediction model for ARDS that 1) uses only structured data automatically collected from the EHR and 2) produces estimates of ARDS risk at relevant time points throughout a patient’s course of illness would be of great clinical value. Such models could be applied prospectively with minimal resources to identify ARDS risk throughout a hospitalization and guide therapeutic interventions. In this study, we developed a machine learning approach to risk stratify patients for ARDS using data that can be automatically extracted from the EHR. Applied to a cohort of critically-ill patients, we hypothesize that a model that leverages EHR data can provide accurate and timely estimates of ARDS risk without requiring manual chart abstraction.

## Materials and methods

**Fig 1:**
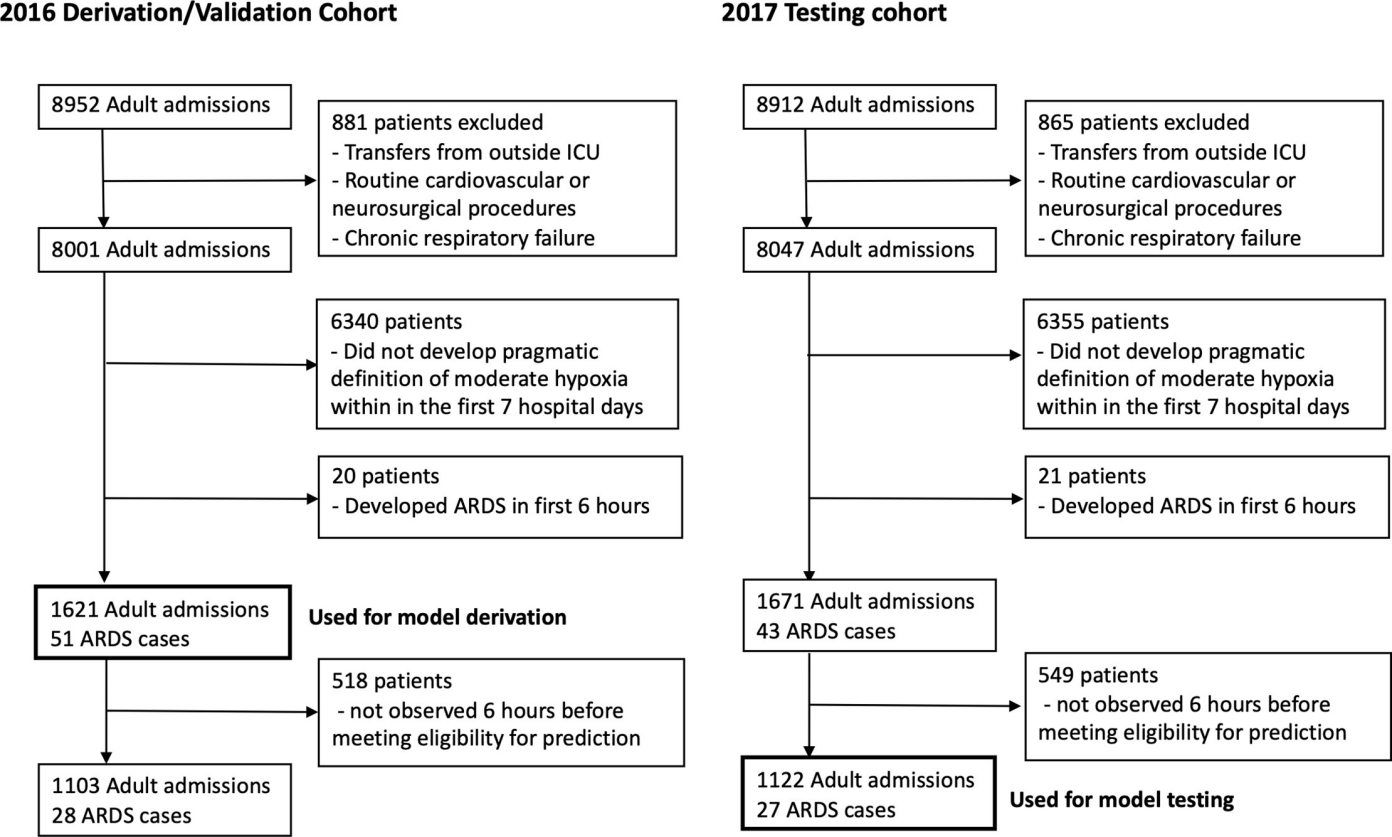
Study cohort. Flow diagram of 2016 model derivation cohort and 2017 testing cohort.

We trained and validated a risk stratification model for ARDS using a cohort of consecutive adult patients (Age ≥ 18) hospitalized at a single, large tertiary care center between January 1 and March 31, 2016. We then tested the model in a temporally distinct cohort of patients hospitalized between January 1 and March 31, 2017 at the same center (Fig 1).

We excluded patients who transferred directly to the ICU from another hospital (because ARDS may have been the reason for transfer), who were admitted to a specialized hospital unit that cares for patients with chronic respiratory failure on home mechanical ventilation, or were admitted after low-risk cardiac or neurosurgical procedures. EHR data were abstracted from the first seven days of hospitalization for all patients. Patients were defined as eligible for ARDS risk stratification if at any point during the first seven days they required more than 3L of supplemental oxygen on two consecutive recordings and had a complete blood count drawn. This pragmatic definition of “moderate” hypoxia helped identify patients at a time when clinicians are likely interested in estimating risk of ARDS. We focused on this seven-day window since the vast majority of ARDS cases occur within the first week of hospitalization [8].

### Generating “Gold Standard” ARDS labels

**Fig 2:**
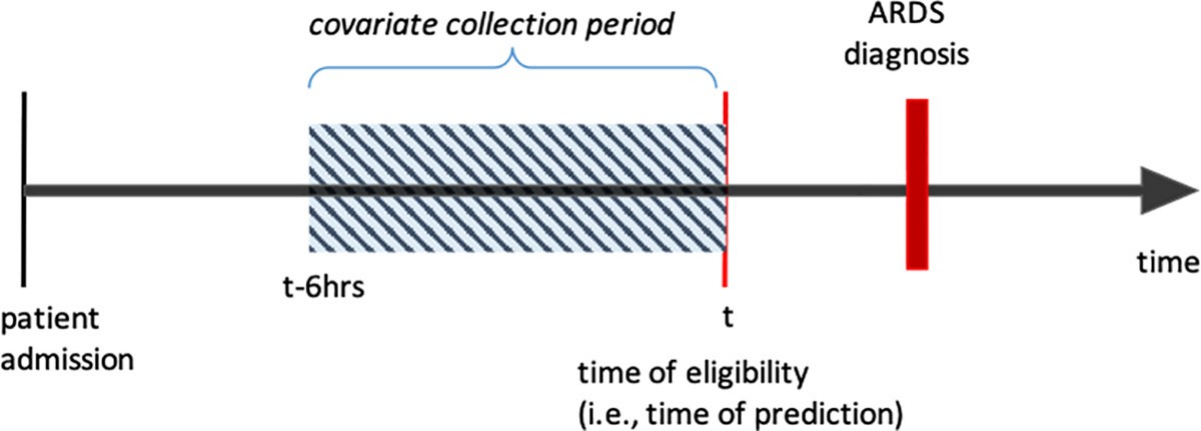
Timeline for prediction. At the patient’s time of eligibility (i.e., when they develop moderate hypoxia), the patient’s risk of future ARDS was predicted using the most recent six hours of data.

Patients in the 2016 and 2017 cohorts were independently reviewed for the development of ARDS by at least two critical care trained physicians if at any point during the first week of hospitalization they had a calculated PaO_2_/FiO_2_ < 300 (or SaO_2_/FiO_2_ < 300) [11] while receiving invasive mechanical ventilation, non-invasive ventilation, or high flow nasal cannula. Because this is the minimal level of hypoxemia required to meet ARDS criteria [12], patients who did not meet this minimum were labeled as not developing ARDS. For patients reviewed for ARDS, physicians determined whether patients developed ARDS and identified the time when all criteria were met based on the Berlin ARDS definition, using a previously published approach [13]. If two physicians disagreed about whether ARDS developed, a third physician reviewed the patient’s chart and the patient was labeled based on the majority result. For patients with ARDS, the minimum time any reviewer felt all ARDS criteria were met was defined as the time of ARDS onset. We excluded patients who developed ARDS in the first six hours of hospitalization, or who met study eligibility criteria at the time ARDS diagnosis, because our interest was in developing a model that could identify patients at greatest risk for ARDS, not those who already developed ARDS (Fig 2)

### Electronic health data extraction and preprocessing

For each patient in our study cohort, we extracted EHR data pertaining to the six hours preceding the time when eligibility criteria were met (Fig 2). We included baseline patient characteristics (e.g., age, race, and sex) and structured, time-stamped data elements (laboratory values, vital signs, medication administration records) from the six-hour window. We focused on structured data elements that could be readily extracted from the EHR in real-time. Thus, we did not include data from unstructured free-text notes or discharge diagnosis codes. Through a series of preprocessing steps, we mapped all clinical data to a vector of binary values (1 or 0). In general, continuous variables were first quantized and mapped to binary feature vectors by dividing the data into quintiles [14]. We transformed continuous variables into categorical variables to capture potential non-linear relationships, while maintaining the simplicity and interpretability of a linear model. For example, age was first discretized into quintiles of the following ranges: 18–35, 35–40, 40–55, 55–68, > 68, then each patient’s age was mapped to a five-dimensional binary feature. Thus, a 66-year old patient would be represented as the five-dimensional feature vector (0, 0, 0, 1, 0).

For numerical variables recorded potentially multiple times within the six-hour window, e.g. heart rate, we computed summary statistics based on all recorded measurements. For each variable, six summary measures were calculated: minimum, maximum, mean, median, standard deviation, interquartile range. Each of these summary statistics was then quantized and mapped to binary variables as described above. Here, the absence of a test result can be informative, i.e., the data are not missing at random. Thus, to capture this potentially important information, we included a “missing” category to encode whether or not a test was not available in the six-hour window [15]. Therefore, a time-varying clinical variable could be mapped to a feature vector as large as 31 dimensions (6 summary statistics x 5 quintiles + 1).

Categorical variables with *d* categories were mapped to a *d*-dimensional vector, where each dimension corresponded to a different category. Laboratory test results were split into categories based on standard reference ranges: “critically high”, “high”, “normal”, “low” and “critically low.” If multiple results of the same laboratory test were available in the six-hour window, all results were used to generate the feature vector. A “missing” category was included and set to 1 if the test was not available in the six-hour window or 0 otherwise. A “count” category was also included to capture the frequency of these laboratory tests. These counts were then quantized and mapped to binary variables as described above. Medications were grouped into categories. If a medication from a specific category was administered within the six-hour window, its corresponding value was set to 1 or 0 otherwise. We excluded any medications specifically related to ARDS treatment (e.g., neuromuscular blockade). Additional details of the EHR pre-processing are described in the methods appendix (S1 appendix).

### Model training, validation, and testing

**Fig 3:**
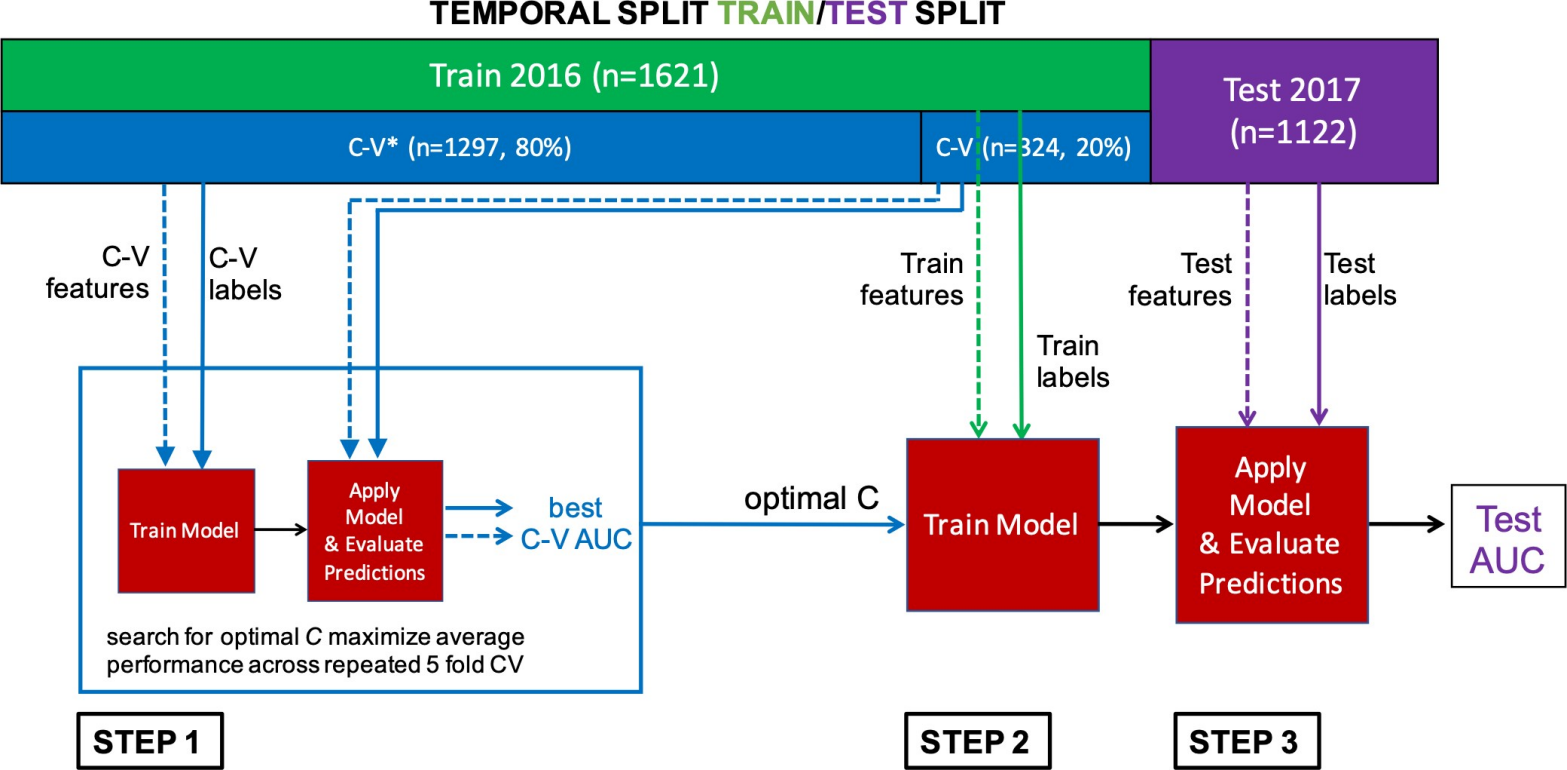
Model training, validation and testing pipeline. The data was split temporally into a training/validation dataset (2016) and testing dataset (2017). Step 1: 5-fold cross validation was performed using the 2016 dataset to identify the optimal model hyperparameter. Step 2: The model was re-trained using the optimal hyperparameter on the entire 2016 dataset to learn model parameters. Step 3: The model was evaluated on held-out test data from 2017.

After EHR data preprocessing, each patient in the 2016 and 2017 cohorts was represented by a final feature vector of 984 dimensions. We split these data into training, validation, and test sets (Figs 1 and 3). Data from 2016 were used for model training and validation, including learning model parameters and hyperparameters (further described below). We then evaluated the model on a temporally distinct dataset of patients admitted in 2017 which served as the test set (Fig 3). Compared to a random split, this more closely mimics how the model would perform in practice when applied in the future and is generally considered a stronger validation design [16].

We used regularized logistic regression to learn a mapping from the 984-dimensional feature space to the probability of developing ARDS. We chose *L*_2_ regularization, to keep all potentially important features. By keeping all features, we were able to more easily identify potential label leakage, i.e. variables indicating that the model had simply learned to identify patients with ARDS rather than predict ARDS (e.g., variables associated with ARDS treatment). For comparison, we also trained an *L*_1_ regularized logistic regression model. L_1_-regularization can lead to a sparser solution (i.e., force many coefficients to zero). In contrast to *L*_2_ regularization, *L*_1_ removes features that are highly correlated with other important features, making it more difficult to identify label leakage. In settings exhibiting high-collinearity, *L*_1_ regularization may drop parameters that are known to affect the outcome of interest [17]. Finally, to explore the potential for non-linear relationships among variables and the outcome of interest, we trained an extreme gradient boosted decision tree model (XGBoost)[18].

Hyperparameters were selected by performing repeated 5-fold cross validation on the 2016 dataset. During repeated cross validation, we used grid search to find the optimal model hyperparameters based on the area under the receiver operating characteristic curve (AUROC). The *L*_2_ and *L*_1_ regularization models both have one hyperparameter, which balances the complexity of the model and the performance on the training set. XGBoost has six hyperparameters to optimize (further details described in the methods supplement S1 Appendix). After we selected the hyperparameters for each model, we then retrained each model (i.e., learning the feature weights) using the entire 2016 dataset (Fig 3).

### Comparison to published ARDS prediction scores

For comparison, we developed an automated version of the Early Acute Lung Injury (EALI) score [19] and evaluated its performance on the 2017 patients. The EALI score is a three-component score that predicts ARDS prior to the need for invasive mechanical ventilation. The score was calculated using the maximum respiratory rate (1 point: respiratory rate > 30) and maximum supplemental oxygen level (1 point: 2–6 L/min, 2 points: > 6 L/min) in the six-hour window prior to prediction eligibility. Immunosuppression (1 point) was defined as chemotherapeutic, steroid (≥ 20 mg prednisone equivalents), or other immunosuppressant administration at any point prior to prediction eligibility. We did not directly compare our model to LIPS as part of our analysis, because it was not possible to retrospectively determine what data required to compute the LIPS score would have been available at prediction time.

### Model evaluation

We evaluated the learned models and the EALI score on the held-out 2017 patients. Patients were excluded from the 2017 test cohort, if they became eligible for prediction within the first six hours of the admission, since in such cases it was not possible to extract the required six hours of data for input to the model.

For each model, we calculated the AUROC and empirical 95^%^ confidence intervals (i.e., the 2.5^th^ and 97.5^th^ percentiles) using 1000 bootstrapped samples from the 2017 test set. For the *L*_2_-regularized model, we selected a risk threshold based on the 85^th^ percentile of ARDS risk, and generated a confusion matrix to calculate sensitivity, specificity and positive predictive value. We also measured model calibration by plotting observed versus predicted ARDS risk. To further evaluate performance, we evaluated model performance by grouping patients based on the length of time between the time of ARDS prediction and ARDS onset: <6 hours, 6–24 hours, and >24 hours. We also compared the model’s sensitivity stratified by ARDS severity based on PaO_2_/FiO_2_: < 100, severe; 100–200, moderate; > 200 mild [12].

In addition to evaluating the *L*_2_ -regularized model’s overall performance, we examined the model’s learned coefficients to understand which features contribute most to the overall risk score of a patient (and check for potential label leakage). We present the features associated with the ten largest positive coefficients (i.e., risk factors) and the ten largest negative coefficients (i.e., protective factors) in the main text and all feature weights in the appendix.

The institutional review board of the University of Michigan approved this study with a waiver of informed consent, as the research posed minimal risk to the welfare of the subjects that participated. The funders of this work had no role in the study design, data collection and analysis, decision to publish, or preparation of this manuscript.

## Results

The initial training cohort included 1,103 patients admitted between January and March 2016 with 28 patients who developed ARDS (Fig 1). Given the small number of patients who developed ARDS in this cohort, we included patients who became eligible for ARDS risk stratification within six hours after presentation during training (despite not being observed for a full 6 hours, see S1 Table for comparison of these groups). This increased the training data to 1,621 patients and 51 who developed ARDS. The test cohort, which included only patients with a full six-hour observation window prior to ARDS risk stratification, included 1,122 patients admitted between January and March 2017 and had an overall ARDS incidence of 2.4% (27 patients) over the first week of hospitalization (Table 1).

**Table 1:** Study population characteristics.

Clinical characteristics	Training/validation	Test cohort
Year	2016	2017
Number (N)	1621	1122
Diagnosed with ARDS	51	27
Median age [IQR]	62 [51–71]	62 [51–72]
Female (%)	45.5	42.9
Race (%)		
Caucasian	85.5	74.4
Black	9.1	8.5
Other	5.4	17.1
Admission Source (%)		
ED	66.3	54.1
Post-op	22	31.6
Floor	11.7	14.4
**Clinical outcomes**		
Length of stay, d median [IQR]	5 [3–9]	6 [3–9]
ARDS onset, hr median [IQR]	43.2 [19.1–72.1]	38.0 [21.5–72.6]
In-hospital mortality (%)	6.0	4.6

**Fig 4:**
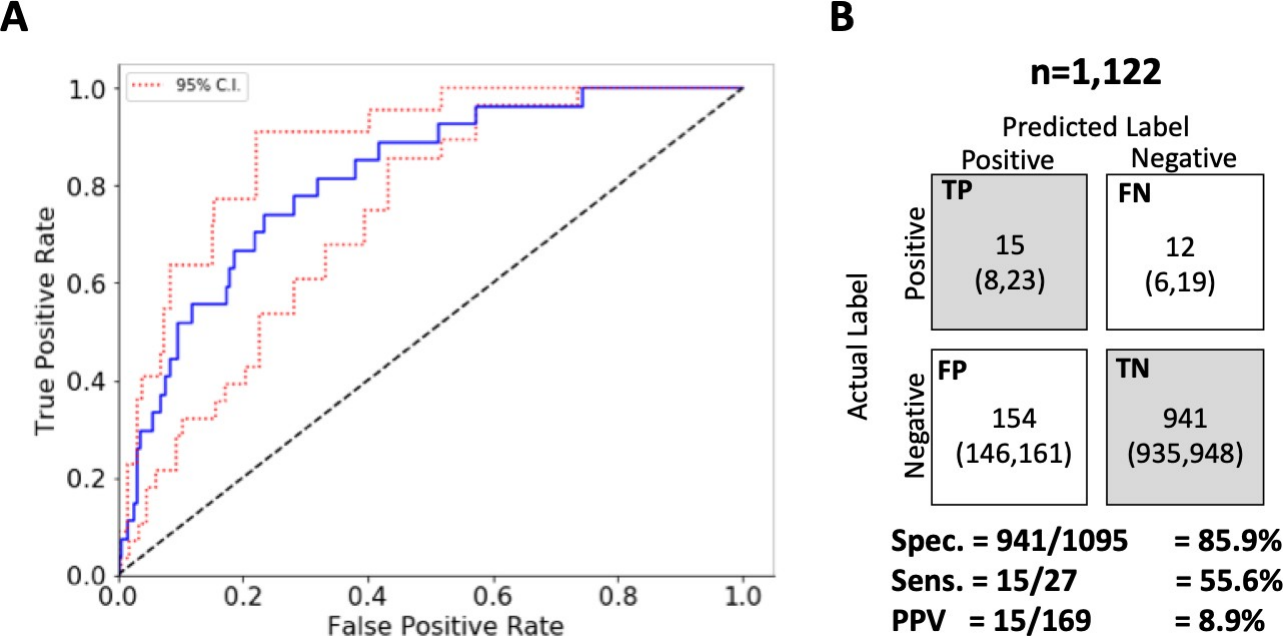
Model Performance. Performance of the ARDS risk prediction model (L2-regulized model) in the 2017 test cohort. A. ROC curve and 95% interval estimates. B. Confusion matrix with 95% interval estimates.

The *L*_2_-regularized logistic regression model demonstrated the best discriminative performance among all the models, achieving an AUROC = 0.81 (95% CI: 0.73–0.88) on the 2017 test cohort (Fig 4A). This was comparable to the discriminative performance on the validation set AUROC = 0.82, suggesting the model is not overfitting despite the large number of features. The *L*_1_-regularized logistic regression model achieved a test AUROC = 0.76 (95% CI: 0.68–0.84), and forced 929 of 984 model coefficients to zero during derivation. Similarly, XGBoost achieved a test AUROC = 0.75 (95% CI: 0.68–0.81). The EALI score had an AUROC = 0.60 (95% CI: 0.50–0.69) on the 2017 dataset.

Selecting a risk threshold based on the 85^th^ percentile of risk, the *L*_2_-regularized logistic regression model had a sensitivity of 56% (95% CI: 35%, 74%), specificity of 86% (95% CI: 85%, 87%) and positive predictive value of 9% (95% CI: 5%, 14%) (Fig 4B). At this risk threshold, the model identified a subpopulation of patients at 4 times the population’s baseline risk. Actual risk increased monotonically with predicted risk, demonstrating fair calibration performance (S1 Fig).

**Fig 5:**
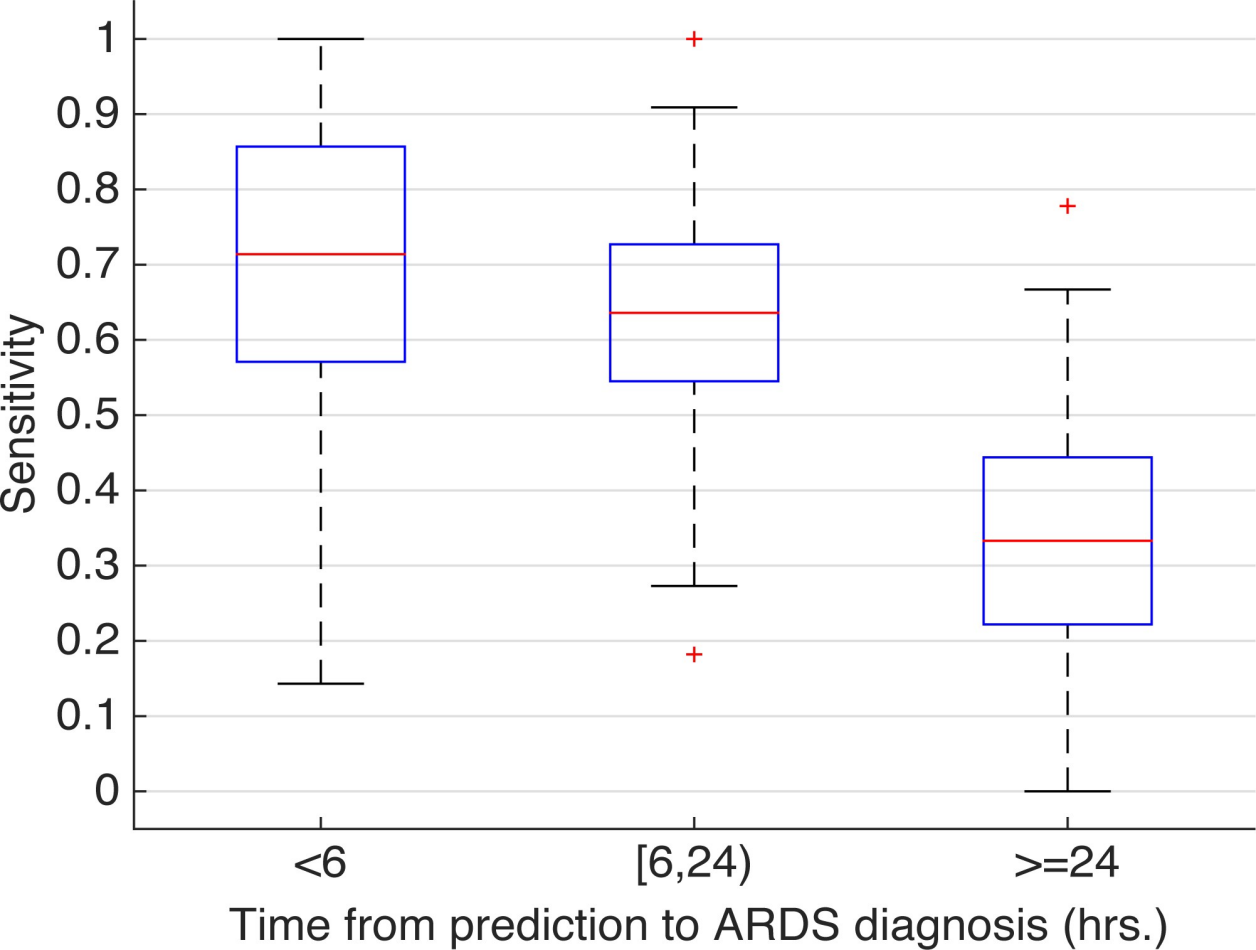
Model Sensitivity stratified by ARDS time of onset. Model performance in subgroups of ARDS patients based on time from ARDS risk stratification to ARDS onset.

We compared model performance across subgroups based on the time from prediction to ARDS onset (Fig 5). The model sensitivity was higher for patients who developed ARDS less than 6 hours after risk stratification compared to patients who developed ARDS over 24 hours after risk stratification, sensitivity of 71% and 34% respectively (though empirical confidence intervals for these estimates are wide). The median time from ARDS risk stratification to ARDS onset for those who developed ARDS was 10 hours (IQR 2–34). Model sensitivity across subgroups based on ARDS severity did not significantly vary (S2 Fig).

We examined the ten predictive (highest weight) and protective (lowest weight) features identified by the model (Table 2 and S2 Table). Measures of hypoxemia, including the minimum calculated PaO_2_/FiO_2_ ratio and the minimum oxygen saturation in the six-hour window prior to risk stratification were predictive of ARDS development. Measures that captured patients with a persistently elevated heart rate, for example, a median heart rate during the six-hour window in the highest quintile, were also predictive. Normal hemoglobin and platelet count also predicted ARDS development. Protective features included the absence of a lactate or pH result, middle quintile of age, and other measures capturing a normal heart rate and oxygen saturation.

**Table 2:** Top predictive factors. Top 10 risk factors and top 10 protective factors identified in the model to risk stratify patients for ARDS.

**Top 10 Risk factors**				
**Rank**	**Description**	**Value range**	**Coefficient**
1	Low minimum PaO2/FiO2^a^	124–161	0.15
2	High minimum heart rate	> 95	0.15
3	Normal hemoglobin level	12–16 g/dL	0.14
4	High albumin level	> 5 g/dL	0.14
5	Low minimum O2 saturation^b^	< 89%	0.13
6	Very high median heart rate	> 104	0.13
7	Very high mean heart rate	> 104	0.12
8	Normal platelet count	150–400	0.12
9	Low Interquartile range systolic BP	0–4	0.12
10	High Standard deviation O2 saturation	1.9–2.9	0.11
**Top 10 Protective factors**				
**Rank**	**Description**	**Value range**	**Coefficient**
1	Missing lactate result	n/a	-0.12
2	Missing pH result	n/a	-0.12
3	Location: scheduled chemotherapy	n/a	-0.12
4	Middle age range	47–58	-0.11
5	Normal bicarbonate	22–34	-0.11
6	Low O2 saturation standard deviation	0.6–1.3	-0.1
7	Low minimum heart rate	65–74	-0.1
8	Low maximum heart rate	48–80	-0.1
9	Very high mean O2 saturation	> 98%	-0.1
10	Middle Mean heart rate	83–92	-0.1

Model features were derived using data from the six hours preceding the time when the patient met eligibility criteria. Summary measures, including minimum value, maximum, mean, median, standard deviation, and interquartile range were calculated for each continuous variable recorded multiple times during the six-hour window (e.g. heart rate). Inter-quartile range is the difference between the 75^th^ and 25^th^ percentile value. These summary measures were then quantized as described in the methods.

^a^PaO2/FiO2 was directly calculated or derived based on recorded O2 saturation of an arterial blood gas measurement was absent [11].

^b^Lowest minimum oxygen saturation may have occurred when the patient was on at least 3 liters of supplemental oxygen at the time of ARDS prediction or in the prior six hours when the patient was on less than this amount of oxygen.

## Discussion

Patient risk stratification tools for ARDS development could enable more timely diagnosis and targeted treatments. However, current approaches rely on manual chart abstraction and make predictions at a single point in time during the admission, limiting their clinical value. We sought a machine learning approach in which the risk predictions could be automated. We learned and evaluated models to risk stratify patients for the development of ARDS, based solely on structured data elements from the EHR. Models were learned (i.e., derived) using patient admissions in 2016 and evaluated on a temporally distinct cohort from 2017. Despite the small sample size, the best performing model achieved good discriminative performance on the held-out test set AUROC = 0.81, identifying patients at four times higher risk of ARDS within the cohort and 17 times the baseline risk of hospitalized adults. For patients who developed ARDS, the model identified them as high risk a median of 10 hours before ARDS onset. This analysis demonstrates the feasibility of a machine learning approach to ARDS risk stratification using automatically extracted EHR data and represents a benchmark for further efforts.

Previous work in ARDS risk stratification centers around the Lung Injury Prediction Score (LIPS). During the LIPS score validation, clinical coordinators enrolled patients if they were identified as having an ARDS risk factor, and then performed a manual chart abstraction to calculate the score. In contrast, our goal was to develop a model that could be fully automated without human chart abstraction. In our approach, patients were identified for risk-prediction (i.e., eligibility) using an EHR-based definition of moderate hypoxia, and the model performed ARDS risk stratification using only structured EHR data. We did not directly compare the model against LIPS in the current study, because it was not possible to accurately determine which of the required LIPS clinical data (e.g., the presence of pneumonia or sepsis) would have been known by clinicians at prediction time by a retrospective chart review.

We did compare the EHR-based ARDS risk stratification model to the EALI score, a three component ARDS risk score that was straight-forward to automate using EHR data. The EALI model had markedly lower performance than the other models in the 2017 patients. This could be due, in part, to fact that the EALI score was originally derived in a highly selective patient population that had been reviewed by a physician for bilateral airspace disease on chest imaging and absence of left atrial hypertension [19], which are important ARDS definition criteria [12]. Additionally, given the relatively small number of variables we would not expect it to perform better than the EHR-based model. Our results suggest that, given the complexity of ARDS, there are many (possibly hundreds) important variables to consider when estimating patient risk of developing ARDS.

The EHR-based ARDS risk stratification model identified predictive clinical factors that have not previously been shown to be associated with the development of ARDS. Given the observational nature of the data and high-collinearity between individual feature weights in the model, definitive conclusions regarding the effect of any one variable should not be made. However, these results are hypothesis generating and warrant further evaluation. Abnormal elevation in heart rate was a positive predictor for ARDS in the model, including elevated minimum and median heart rate in the six-hour window prior to prediction. As heart rate is a key marker to systemic inflammatory response, this association could be indicative of an early manifestation of the inflammatory response leading to ARDS [20]. Alternatively, the elevated heart rate may be a normal physiologic response to worsening hypoxemia. Both a normal platelet count and hemoglobin level were associated with increased ARDS risk in the model. There are several postulated mechanisms by which platelets and hemoglobin may contribute to the development of acute lung injury and ARDS [21, 22]. Missing pH and lactate level, both negative predictive factors, may not have been performed because the treating clinicians were not concerned that the patient was severely ill at the time of ARDS prediction.

While our model had good discriminative performance, at a threshold of the 85^th^ percentile of risk, the model had a sensitivity of 56% and positive predictive value (PPV) of 9%, identifying a population of patients at 4 times the cohort’s baseline ARDS risk. The PPV implies that approximately 1 out of every 11 patients identified as high-risk by the model will actually develop ARDS. In settings where the baseline prevalence is very low <2.5% a PPV of 9% is clinically useful assuming a low-cost, low-risk intervention. Depending on the specific clinical need, this threshold could be further tuned, resulting in a change to the model’s sensitivity and positive predictive value. For example, while the current threshold may be appropriate for considering ICU transfer, a higher threshold and resulting higher positive predictive value may be necessary when considering a treatment with potential harms. This model could be applied continuously if such a scenario was necessary, potentially still identifying patients prior to ARDS, but at a time point closer to ARDS onset.

The current model represents a significant step towards building tools for automatically identifying patients at greatest risk of developing ARDS. There are a number of interesting directions in which others could build upon this work. These include methods that incorporate variable length inputs e.g., long short-term memory (LSTM) networks [23, 24]. While, such methods currently lack interpretability (i.e., are black boxes), which may be less appropriate in high-stakes settings like healthcare, researchers are currently working on techniques to improve transparency of such approaches. As the relatively poor performance of the XGBoost model demonstrated, we likely do not yet have enough examples to learn robust non-linear models. However, as we continue to amass training examples, such non-linear techniques could lead to further improvements in predictive performance. As an alternative to the labor-intensive process of collecting ground truth labels, one could augment the current approach with a semi-supervised approach, taking advantage of the large quantities of EHR data now available.

Our analysis has other limitations. As the model was derived using single-center data, it is not known whether the learned relationship between model’s features and risk of ARDS would generalize to other institutions. Additional work is needed to understand how EHR-based prediction models derived at one institution perform at other institutions. To make such comparisons easier, our Appendix includes all the individual features and feature weights to allow transparent comparisons. The current analysis presents an approach that can be used to derive institution-specific risk stratification models. In our previous work, we have shown how models can be more predictive when they are tailored to a specific patient population and health centers [15]. Practice variation or differences in EHR documentation may lead to differences in the performance of EHR-based models across institutions or populations. While a more tailored model is likely to perform better at a specific institution, the additional work required to develop and maintain such a model is an important trade-off that needs consideration as models become more ubiquitous in clinical practice.

## Conclusion

We developed an ARDS risk prediction model based on EHR data with good discriminative performance. Our results demonstrate the feasibility of a data-driven approach to ARDS risk stratification solely from data that can be extracted automatically from the EHR. Deployed clinically, such models could identify patients at high risk for the development of ARDS and enable new approaches to ARDS treatment, including therapies that prevent or halt the progression of ARDS at its earliest manifestations.

## Supporting information

S1 STROBE checklist cohort (DOC)

S1 Appendix Methods appendix. (DOCX)

S1 Table Characteristics of the 2016 training/validation cohort and 2017 test cohort. (DOCX)

S2 Table Top positive and negative feature weights with coefficient values and 95% interval estimates. (DOCX)

S1 Fig Calibration curve for the 2017 test set. (DOCX)

S2 Fig Model sensitivity based on ARDS severity. (DOCX)

S3 Fig Feature Elimination. (DOCX)

S1 Model Features (XLSX)
