# Supplementary material for: Machine learning for patient risk stratification for acute respiratory distress syndrome
Source: PLoS One. 2019 Mar 28;14(3):e0214465. doi: 10.1371/journal.pone.0214465 (PMC6438573; doi:10.1371/journal.pone.0214465)
Supplement: S1 STROBE checklist cohort — (DOC) [file pone.0214465.s001.doc]

STROBE Check list for the study: Machine Learning for Patient Risk Stratification for Acute Respiratory Distress Syndrome

|  | Item No | Recommendation |
| --- | --- | --- |
| **Title and abstract** | 1 | (*a*) Indicate the study’s design with a commonly used term in the title or the abstract  Page 1. Title: Machine Learning for Patient Risk Stratification of Acute Respiratory Distress Syndrome |
|  |  | (*b*) Provide in the abstract an informative and balanced summary of what was done and what was found  Page 2. Abstract |
| Introduction | | |
| Background/rationale | 2 | Explain the scientific background and rationale for the investigation being reported  Page 3, line 44 – 59, introduction |
| Objectives | 3 | State specific objectives, including any prespecified hypotheses  Page 4, line 66, hypothesis: “we hypothesize that a model that leverages EHR data could achieve good discriminative performance while also having the flexibility of producing estimates of risk without manual chart abstraction” |
| Methods | | |
| Study design | 4 | Present key elements of study design early in the paper  Page 4, line 71-74, cohort study |
| Setting | 5 | Describe the setting, locations, and relevant dates, including periods of recruitment, exposure, follow-up, and data collection  Page 4, line 71-74, single tertiary care center, patients hospitalized Jan-March 2016 and Jan-March 2017 |
| Participants | 6 | (*a*) Give the eligibility criteria, and the sources and methods of selection of participants. Describe methods of follow-up  Page 5, line 76-84, and Figure 1 |
|  |  | (*b*) For matched studies, give matching criteria and number of exposed and unexposed  n/a |
| Variables | 7 | Clearly define all outcomes, exposures, predictors, potential confounders, and effect modifiers. Give diagnostic criteria, if applicable  Outcome: Page 5, line 87 – 96, Generating the “Gold Standard” ARDS labels;  Predictors: Page 6, line 114 – Page 7, line 133 |
| Data sources/ measurement | 8* | For each variable of interest, give sources of data and details of methods of assessment (measurement). Describe comparability of assessment methods if there is more than one group  Predictors: Page 6, line 114 – Page 7, line 133 |
| Bias | 9 | Describe any efforts to address potential sources of bias  Page 7, line 136 – Page 8, line 146 |
| Study size | 10 | Explain how the study size was arrived at  n/a |
| Quantitative variables | 11 | Explain how quantitative variables were handled in the analyses. If applicable, describe which groupings were chosen and why  Page 6, line 113-119 |
| Statistical methods | 12 | (*a*) Describe all statistical methods, including those used to control for confounding  Page 7, line 136 – Page 8, line 170 |
|  |  | (*b*) Describe any methods used to examine subgroups and interactions  Subgoups of ARDS severity and subgroups of time from prediction to ARDS onset |
|  |  | (*c*) Explain how missing data were addressed  Page 7, line 126 |
|  |  | (*d*) If applicable, explain how loss to follow-up was addressed  n/a |
|  |  | (*e*) Describe any sensitivity analyses  n/a |
| Results | | |
| Participants | 13* | (a) Report numbers of individuals at each stage of study—eg numbers potentially eligible, examined for eligibility, confirmed eligible, included in the study, completing follow-up, and analysed  Page 8, line 181 – 188, Figure 1. Study flow diagram |
|  |  | (b) Give reasons for non-participation at each stage  Figure 1. Study flow diagram |
|  |  | (c) Consider use of a flow diagram  Figure 1. Study flow diagram |
| Descriptive data | 14* | (a) Give characteristics of study participants (eg demographic, clinical, social) and information on exposures and potential confounders  Table 1 |
|  |  | (b) Indicate number of participants with missing data for each variable of interest  n/a |
|  |  | (c) Summarise follow-up time (eg, average and total amount)  Page 5, line 80 |
| Outcome data | 15* | Report numbers of outcome events or summary measures over time  Table 1 |
| Main results | 16 | (*a*) Give unadjusted estimates and, if applicable, confounder-adjusted estimates and their precision (eg, 95% confidence interval). Make clear which confounders were adjusted for and why they were included  Page 11 |
|  |  | (*b*) Report category boundaries when continuous variables were categorized  Page 11 |
|  |  | (*c*) If relevant, consider translating estimates of relative risk into absolute risk for a meaningful time period  n/a |
| Other analyses | 17 | Report other analyses done—eg analyses of subgroups and interactions, and sensitivity analyses  Page 12 |
| Discussion | | |
| Key results | 18 | Summarise key results with reference to study objectives  Page 12, line 240 - 251 |
| Limitations | 19 | Discuss limitations of the study, taking into account sources of potential bias or imprecision. Discuss both direction and magnitude of any potential bias  Page 14 |
| Interpretation | 20 | Give a cautious overall interpretation of results considering objectives, limitations, multiplicity of analyses, results from similar studies, and other relevant evidence  Page 17 |
| Generalisability | 21 | Discuss the generalisability (external validity) of the study results  Page 17 |
| Other information | | |
| Funding | 22 | Give the source of funding and the role of the funders for the present study and, if applicable, for the original study on which the present article is based  Including in a separate location of the submission per PLOS: Medicine guidelines |

*Give information separately for exposed and unexposed groups.

**Note:** An Explanation and Elaboration article discusses each checklist item and gives methodological background and published examples of transparent reporting. The STROBE checklist is best used in conjunction with this article (freely available on the Web sites of PLoS Medicine at http://www.plosmedicine.org/, Annals of Internal Medicine at http://www.annals.org/, and Epidemiology at http://www.epidem.com/). Information on the STROBE Initiative is available at http://www.strobe-statement.org.
