# Supplementary material for: Machine learning for patient risk stratification for acute respiratory distress syndrome
Source: PLoS One. 2019 Mar 28;14(3):e0214465. doi: 10.1371/journal.pone.0214465 (PMC6438573; doi:10.1371/journal.pone.0214465)
Supplement: S1 Appendix — (DOCX) [file pone.0214465.s002.docx]

**S1 Appendix. Methods Appendix**

Recursive binning strategy

This strategy was employed as a pre-processing step to handle all the continuous-valued variables in the dataset. The algorithm bins the values of a variable into a maximum of $n=5$ bins (quintiles) and if the distribution of the values makes that impossible or counter-intuitive (e.g., if 40% of the values are the same or lie in one bin it would not make sense to split this bin into 2), then we attempt to bin the values into n-1 bins, repeating until the values can be cleanly separated by some number of quantiles. Quintiles were chosen since they have been proven to capture the distribution of the data better than other binning method

Naming convention of supplementary file of model features (S2_model_features)

All numerical features usually have 4 components, each separated by ‘_’. The first component is the name of the feature itself (e.g., ageinyears, hr, rr). The second component is the summary statistic in question (e.g., min, max, mean). The third is the particular bin they fall into represented as (x,y] where x and y represent the left and right boundaries of the bin. The fourth component represents the bin number and the total number of bins. For example, the column: ’ageinyears_(58.0,65.0]_b3_t5’ refers to the number of patients whose age (in years) falls between 58 and 65, which represents the third bin of the total 5 quintiles. Finally, any column with a ‘_missing’ appended, represents whether that patient had a missing reading. The lab features are represented as the lab test conducted followed by the flag level recorded.

**Details of the clinical variable preprocessing and features in the model**

| **Variable Category** | **Example** | **Pre-processing** | **Number of Variables** | **Total number of features** |
| --- | --- | --- | --- | --- |
| **Baseline Numerical** | Age | Recursive binning | 1 | 5 |
| **Baseline Categorical** | Gender, Race | Binarized by category | 11 | 21 |
| **Laboratory Test Flags*** | pH Very High | Binarized by flag levels | 21 | 168 |
| **Continuous Variables** | Heart rate | Recursive binning after summarization | 32 | 664 |
| **Medication Data*** | Clindamycin, Albumin | Binarized by administration | 127 | 127 |
| **TOTAL** | | | **190** | **985** |

Training the Extreme Gradient Boosting Decision Tree Model (XGBoost)

We performed a grid search during repeated cross validation on the 2016 data to find the optimal values of the six hyperparameters in the XGBoost model. These parameters control the complexity of the model, and include the number of boosted trees, ‘n_estimators,’ the maximum depth of a tree, ‘max_depth’, and the minimum number of instances in each node, ‘min_child_weight’. The ‘max_depth’ can be interpreted as the maximum number of different features that our model can use. Additional parameters included ‘gamma’ or the regularization parameter and best ‘subsample’ and ‘colsample_bytree’ rates which determine the fraction of each tree row and column, respectively, that is used to build the final model.

Feature Elimination

A backwards feature elimination can be used to explore the importance of individual features and identify the smaller subset of features that can be used for risk stratification. However, given the dimensionality of our feature set, this approach is prohibitively expensive. So instead, we examined the performance of the model on the test set, if we eliminated features with relatively small coefficients. That is, we ranked all features according to the absolute value of their model coefficients and then eliminated features with the smallest coefficients, one by one, based on this ranking. At each step, we applied the model to the test set and plotted the results (S3 Fig).
