# Supplementary material for: Machine learning for patient risk stratification for acute respiratory distress syndrome
Source: PLoS One. 2019 Mar 28;14(3):e0214465. doi: 10.1371/journal.pone.0214465 (PMC6438573; doi:10.1371/journal.pone.0214465)
Supplement: S1 Table — (DOCX) [file pone.0214465.s003.docx]

**S1 Table**. **Characteristics of the 2016 training/validation cohort and 2017 test cohort**

| **Clinical characteristics** | **Training/Validation cohort** | | **Test cohort** |
| --- | --- | --- | --- |
| Year | 2016 | 2016 | 2017 |
| Eligible for ARDS prediction | < 6 hours | > 6 hours | > 6 hours |
| Number (N) | 518 | 1103 | 1122 |
| Diagnosed with ARDS | 23 | 28 | 27 |
| Median age [IQR] | 53.5 [53-73] | 61 (50-70) | 62 (51-72) |
| Female (%) | 43.4 | 46.4 | 42.9 |
| Race (%) |  |  |  |
| Caucasian | 83.4 | 83.9 | 74.4 |
| Black | 10.2 | 8.3 | 8.5 |
| Other | 6.4 | 7.9 | 17.1 |
| Admission Source (%) |  |  |  |
| ED | 91 | 55 | 54.1 |
| Post-op | 4.6 | 30.3 | 31.6 |
| Floor | 4.6 | 14.7 | 14.4 |
| **Clinical outcomes** |  |  |  |
| Length of stay, d  median [IQR] | 5 [3-8] | 5 [3-10] | 6 [3-9] |
| ARDS onset, hr  median [IQR] | 21.6 [8.4 - 46.4] | 63.5 [40.8-83] | 38.0 [21.5-72.6] |
| In-hospital mortality (%) | 9.3 | 4.5 | 4.6 |
