# Supplementary material for: Machine learning for patient risk stratification for acute respiratory distress syndrome
Source: PLoS One. 2019 Mar 28;14(3):e0214465. doi: 10.1371/journal.pone.0214465 (PMC6438573; doi:10.1371/journal.pone.0214465)
Supplement: S2 Table — (DOCX) [file pone.0214465.s004.docx]

**S2 Table**. **Top positive and negative feature weights with coefficient values and 95% interval estimates**

**Top 10 Predictive features**

| Rank | Description | Value range | Median coefficient  (95% CI) |
| --- | --- | --- | --- |
| 1 | Minimum PaO2/FiO2 (derived*) | (124,161] | 0.15 (0.07, 0.24) |
| 2 | Minimum heart rate | (95,143] | 0.15 (0.06, 0.22) |
| 3 | Normal hemoglobin | (12, 16) | 0.14 (0.07, 0.21) |
| 4 | High albumin | (5, max) | 0.14 (0.07, 0.22) |
| 5 | Minimum O2 saturation | (0, 89] | 0.13 (0.05, 0.21) |
| 6 | Median heart rate | (104, 176] | 0.13 (0.05, 0.21) |
| 7 | Mean heart rate | (104, 164] | 0.12 (0.04, 0.19) |
| 8 | Normal platelet count | (150, 400) | 0.12 (0.04, 0.20) |
| 9 | Interquartile range systolic BP | (0, 4.0] | 0.12 (0.04, 0.20) |
| 10 | Standard deviation O2 saturation | (1.9, 2.9] | 0.11 (0.01, 0.20) |

**Top 10 Protective features**

| Rank | Description | Value range | Median coefficient  (95% C.I.) |
| --- | --- | --- | --- |
| 1 | Missing lactate result | n/a | -0.12 (-0.19, -0.05) |
| 2 | Missing pH result | n/a | -0.12 (-0.19, -0.05) |
| 3 | hospital location: chemotherapy ward | n/a | -0.12 (-0.20, -0.03) |
| 4 | Age range | (47, 58] | -0.11 (-0.17, -0.05) |
| 5 | Normal bicarbonate | (22, 34) | -0.11 (-0.19, -0.03) |
| 6 | O2 saturation standard deviation | (0.6, 1.3] | -0.10 (-0.15, -0.06) |
| 7 | Minimum heart rate | (65, 74] | -0.10 (-0.15, -0.05) |
| 8 | Maximum heart rate | (48, 80] | -0.10 (-0.14, -0.06) |
| 9 | Mean O2 saturation | (98, 100] | -0.10 (-0.13, -0.06) |
| 10 | Mean heart rate | (83, 92] | -0.10 (-0.15, -0.05) |

*PaO2/FiO2 derived based on recorded O2 saturation

Derived continuous variables using 6 hours of EHR records, e.g. mean or minimum heart rate, were quantized as quintiles unless otherwise stated, laboratory results were divided into 4 categories (low, normal, high, missing) or 6 categories (critically low, low, normal, high, critically high, missing) based on standard reference ranges.
