# Supplementary material for: Machine learning for patient risk stratification for acute respiratory distress syndrome
Source: PLoS One. 2019 Mar 28;14(3):e0214465. doi: 10.1371/journal.pone.0214465 (PMC6438573; doi:10.1371/journal.pone.0214465)
Supplement: S1 Fig — (DOCX) [file pone.0214465.s005.docx]

**S1 Fig**. **Calibration curve for the 2017 test set**
