# Supplementary material for: Machine learning for patient risk stratification for acute respiratory distress syndrome
Source: PLoS One. 2019 Mar 28;14(3):e0214465. doi: 10.1371/journal.pone.0214465 (PMC6438573; doi:10.1371/journal.pone.0214465)
Supplement: S2 Fig — (DOCX) [file pone.0214465.s006.docx]

**S2 Fig. Model sensitivity based on ARDS severity.** Model performance across select subgroups based on severity defined by PaO2/FiO2 at ARDS onset (Mild n = 5, Moderate n = 15, Severe n = 7).
