# Supplementary material for: Machine learning for patient risk stratification for acute respiratory distress syndrome
Source: PLoS One. 2019 Mar 28;14(3):e0214465. doi: 10.1371/journal.pone.0214465 (PMC6438573; doi:10.1371/journal.pone.0214465)
Supplement: S3 Fig — (DOCX) [file pone.0214465.s007.docx]

**S3 Fig. Feature Elimination.** Performance when features are sequentially removed from the EHR-derived risk stratification model (L2 logistic regression model).

**
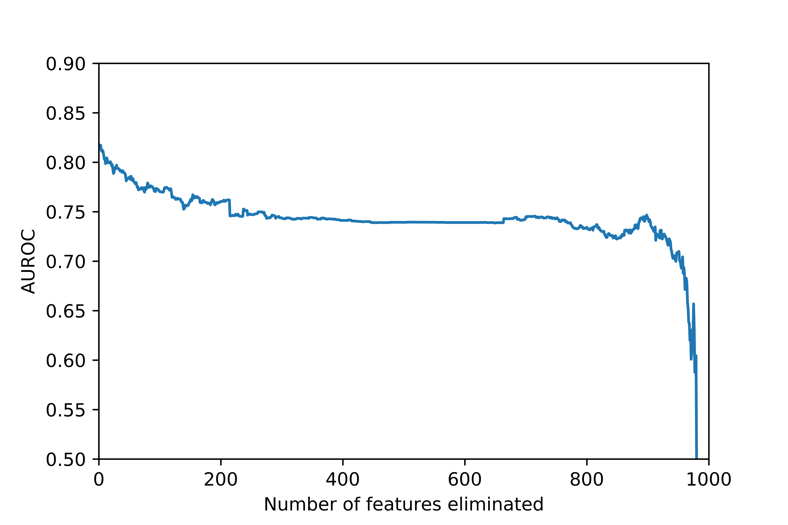
**
